# Long-term persistence of monotypic dengue transmission in small size isolated populations, French Polynesia, 1978-2014

**DOI:** 10.1371/journal.pntd.0008110

**Published:** 2020-03-06

**Authors:** Yoann Teissier, Richard Paul, Maite Aubry, Xavier Rodo, Carlos Dommar, Henrik Salje, Anavaj Sakuntabhai, Bernard Cazelles, Van-Mai Cao-Lormeau

**Affiliations:** 1 Laboratoire de recherche sur les maladies infectieuses à transmission vectorielle, Institut Louis Malardé, Papeete, Tahiti, French Polynesia; 2 Université Paris Descartes, PSL University, Paris, France; 3 Institut Pasteur, Unité de Génétique Fonctionnelle des Maladies Infectieuses, UMR 2000 CNRS, Paris, France; 4 Pasteur Kyoto International Joint Research Unit for Integrative Vaccinomics, Kyoto, Japan; 5 ICREA, Barcelona, Spain; 6 CLIMA (Climate and Health) Program, ISGlobal, Barcelona, Spain; 7 Institut Pasteur, Mathematical Modelling of Infectious Diseases Unit, UMR 2000, Centre National de la Recherche Scientifique, Paris, France; 8 International Center for Mathematical and Computational Modeling of Complex Systems (UMMISCO), UMI 209, Sorbonne Université - IRD, Bondy cedex, France; 9 iGLOBE, UMI CNRS 3157, University of Arizona, Tucson, Arizona, United States of America; 10 IBENS, UMR 8197 CNRS-ENS Ecole Normale Supérieure, Paris, France; London School of Hygiene & Tropical Medicine, UNITED KINGDOM

## Abstract

Understanding the transition of epidemic to endemic dengue transmission remains a challenge in regions where serotypes co-circulate and there is extensive human mobility. French Polynesia, an isolated group of 117 islands of which 72 are inhabited, distributed among five geographically separated subdivisions, has recorded mono-serotype epidemics since 1944, with long inter-epidemic periods of circulation. Laboratory confirmed cases have been recorded since 1978, enabling exploration of dengue epidemiology under monotypic conditions in an isolated, spatially structured geographical location. A database was constructed of confirmed dengue cases, geolocated to island for a 35-year period. Statistical analyses of viral establishment, persistence and fade-out as well as synchrony among subdivisions were performed. Seven monotypic and one heterotypic dengue epidemic occurred, followed by low-level viral circulation with a recrudescent epidemic occurring on one occasion. Incidence was asynchronous among the subdivisions. Complete viral die-out occurred on several occasions with invasion of a new serotype. Competitive serotype replacement has been observed previously and seems to be characteristic of the South Pacific. Island population size had a strong impact on the establishment, persistence and fade-out of dengue cases and endemicity was estimated achievable only at a population size in excess of 175 000. Despite island remoteness and low population size, dengue cases were observed somewhere in French Polynesia almost constantly, in part due to the spatial structuration generating asynchrony among subdivisions. Long-term persistence of dengue virus in this group of island populations may be enabled by island hopping, although could equally be explained by a reservoir of sub-clinical infections on the most populated island, Tahiti.

## Author summary

Dengue virus is the most significant arthropod-borne virus infecting man. Understanding how long dengue virus can persist in populations of varying size is key to understanding its epidemiology. This is, however, impossible to achieve in settings where dengue is endemic, because of continued human movement and is further complexified by the occurrence of several co-circulating serotypes. By contrast, French Polynesia, an isolated group of 72 inhabited islands in the South Pacific, has had intermittent majoritarily monotypic dengue epidemics since the 1940s and offers a unique opportunity to address questions of viral persistence, turnover and the importance of spatial sub-structure in determining dengue epidemiology. Collating and analyzing a database of laboratory-confirmed dengue cases from across French Polynesia over a 35 year period we were able to show that dengue virus die-out can occur with or without replacement by a new serotype, monotypic transmission of dengue viruses fails to be maintained within small island populations but can persist for years among isolated islands connected via air and sea links. This remarkable long-term persistence of dengue virus in French Polynesia could be maintained by asynchronous viral transmission among connected islands and/or by repeated seeding from a reservoir of sub-clinical infections in the most populated island, Tahiti.

## Introduction

Dengue is caused by any of four antigenically distinct dengue virus serotypes, designated DENV-1, DENV-2, DENV-3, and DENV-4, which are transmitted by *Aedes (Ae*.*)* mosquito species. Dengue has become a major international public health concern and over the past decade the number of outbreaks has escalated and the population at risk is increasing yearly [1–4]. More than 3.5 billion people are at risk of DENV infection and it has been estimated that there are 390 million DENV infections every year [5].

Understanding the spatio-temporal dynamics of dengue epidemiology is key for identifying reservoirs of infection, chains of transmission and quantifying the force of infection. Dengue epidemics occur intermittently, driven by climate suitability impacting mosquito density and vectorial capacity, and a sufficient non-immune population. In hyperendemic mainland regions, where the majority of the burden of disease occurs, dengue epidemiology is complexified by the co-circulation and/or rapid sequential circulation of several serotypes, large scale movement of people on daily, monthly and annual timescales, leading to a changing viral community on a changing human background. Coupled with difficulties in differentiating among DENV serotypes using serological methods and the high but variable incidence of inapparent infections, characterizing the spatio-temporal dimension of dengue epidemiology in such settings is challenging [6–9].

In contrast to such mainland hyperendemic settings, French Polynesia has a small population size (275 918 inhabitants, 2017 census) distributed on 72 isolated islands with a climate permissive for perennial mosquito-borne virus transmission [10]. Since 1944, French Polynesia Public Health authorities have reported 14 monotypic dengue epidemics often followed by long periods of low-level transmission and one recent heterotypic epidemic in 2013/4 [11]. Such a scenario offers a unique opportunity to address questions of viral persistence, turnover and the importance of spatial sub-structure in determining dengue epidemiology in small sized geographically isolated populations. Implicit in this first description of the data is the question as to whether French Polynesia is effectively a meta-population whereby DENV is maintained by island-hopping or whether there is a single major source of infection on the main island of Tahiti that subsequently feeds DENV into the smaller islands. We provide here the first collation, description and analysis of dengue dynamics in French Polynesia over a 35-year period, from 1978 to 2014.

## Methods

### Study site, demographic and migration data

**Fig 1:**
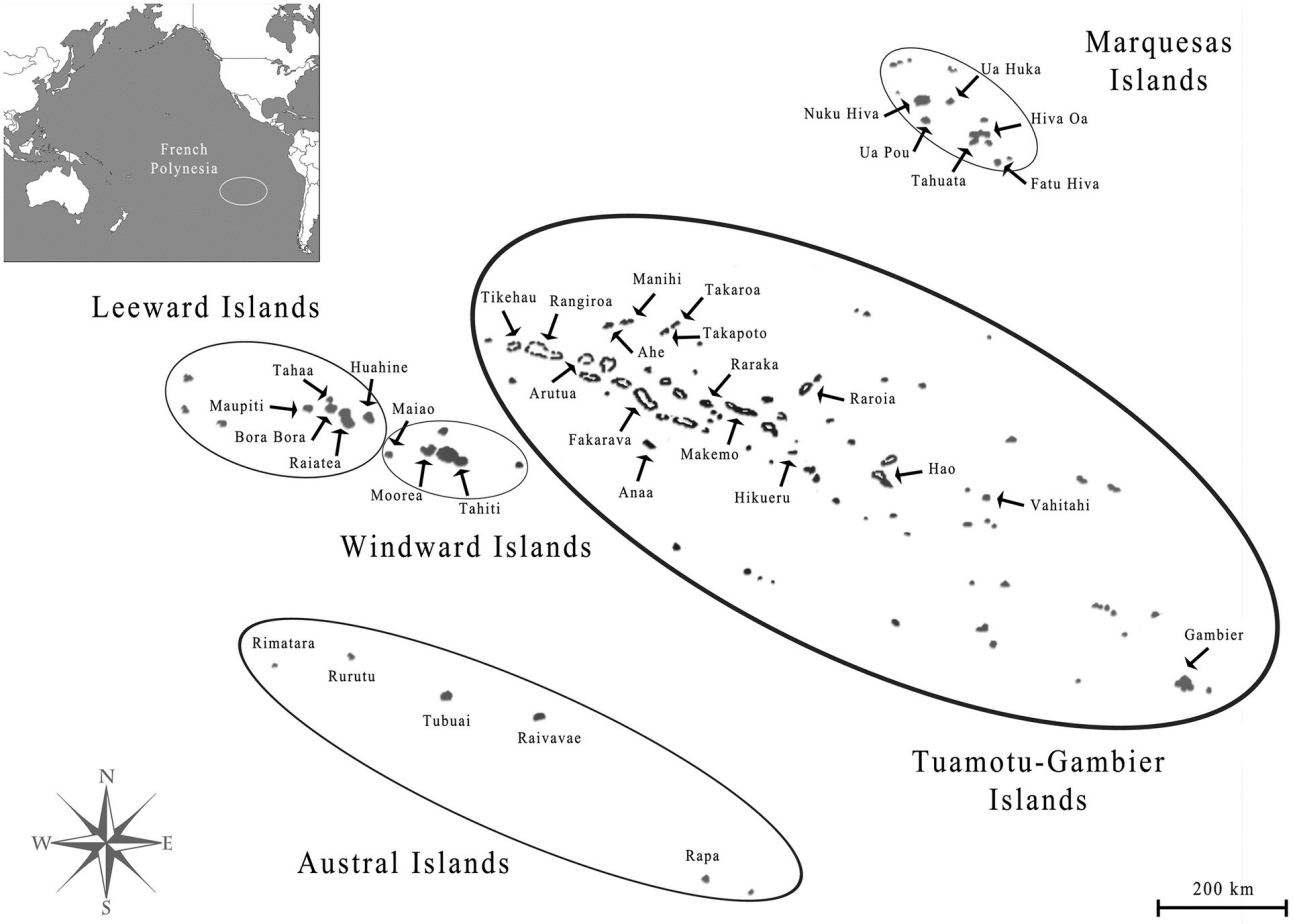
Map of French Polynesia showing the five administrative subdivisions and associated islands. [12].

French Polynesia is located in the middle of the Pacific ocean and is composed of 117 islands [10], grouped into five administrative subdivisions: the Windward Islands (five islands), the Leeward Islands (nine), the Marquesas Islands (12), the Austral Islands (seven) and the Tuamotu-Gambier Islands (68 and 16, respectively)(Fig 1). Out of the 72 inhabited islands, only 15 have a population greater than 1 000 individuals, four (Moorea, Raiatea, Bora-Bora and Tahiti) have more than 10 000 and only Tahiti numbers over 100 000; at the last 2017 census, 68.7% of the total population of 275 918 lived on Tahiti [10]. Island population sizes were taken from the census records from 1971, 1983, 1988, 1996, 2002, 2007 and 2012 [10]. Among island air migration data was obtained from the Service d’Etat de l’Aviation civile en Polynésie Française, Air Tahiti and the Direction de l’aviation civile. Data from 2010 was used for the correlation with the cross-correlation function.

### Dengue case data

Institut Louis Malardé (ILM) is a local biomedical research institute that has been involved in arbovirus diagnostic, surveillance and research in French Polynesia since the 1960s. The ILM receives samples from medical practitioners, health centers and laboratories all over French Polynesia and has recorded dengue confirmed cases from March 1975. The geolocation of the cases only started in August 1978. From 1978 to 2014, different laboratory techniques have been used to confirm dengue cases and, whenever possible, identify the serotype involved: inhibition of haemagglutination assay (1975–1988); intrathoracic inoculation of mosquitoes with the serum from suspected cases (1979–1980); isolation of DENV on C6/36 mosquito cell culture (1984–2005); IgM ELISA (from 1986); Reverse transcriptase Polymerase Chain Reaction (since 2000); NS1 antigen ELISA (from 2006).

### Statistical analyses

Statistical analyses of the distribution of dengue cases were performed by fitting generalised linear models (GLM) to count data (loglinear regression) and proportion positive data (logistic regression). A dispersion parameter was estimated to account for any overdispersion of the data. Cross-correlation functions (CCF) for monthly dengue incidence (per 1000 individuals) across the entire 435 month period (August 1978 to October 2014) were calculated pairwise between the “capital” and the minor islands within each subdivision. Capital islands of each subdivision are defined as those being both highly inhabited and most connected to the other islands by air and boat. Only islands with six months or more incidence of dengue were analysed; thus 21 islands were included. In all cases, the capital islands of each subdivision were taken as the lead island. Then, because islands may be indirectly connected, we repeated the analysis with Tahiti and all other islands. We first examined whether there significant autocorrelation within each island that might generate spurious results. There was no significant autocorrelation within any island within the selected lag month period of 20 months used in the correlation analysis. CCFs were then plotted against log10 population migration between island pairs, using the 2010 air travel data. Absolute air travel volume has altered across the 35-year period but not relatively so across islands.

The sample cross-correlation was calculated between the first island incidence series x and the second island incidence series y lagged at k months using

$$r_{k}=(1-\frac{k}{n}).\;\frac{C_{k}}{\left(S_{x}.S_{y}\right)}$$

Where $C_{k}=\frac{1}{n_{k}}\;\sum_{t=1}^{n-k}(x_{t}-\overset{-}{x})(y_{t+k}-\overset{-}{y})$.

The degree of significance, S, is calculated using a χ^2^ with the number of lag months as the degrees of freedom (here selected as 20).

$$S=n\;\sum_{k=1}^{m}r_{k}^{2}$$

Analyses were carried out in Genstat version 15 [13].

### Wavelet analyses

As dengue dynamics in French Polynesia are very non-stationary, wavelet analyses were performed to explore dengue incidence periodicity [14,15]. Wavelet analysis enables investigation and quantification of the temporal evolution of time series with different rhythmic components. The Morlet wavelet was used and all analyses were performed with Matlab software [14]. All dengue incidence time series were square root transformed and all series were normalized and the trend was suppressed before the computation of the wavelet power spectrum by removing the periodic components greater than 16 years by using a classical low pass filter [14]. Before investigating the synchrony between islands, we applied wavelet clustering [16] to quantify the similarity between the wavelet power spectra of all time series. This approach built cluster trees that grouped the wavelet power spectra based on the similarity of their time–frequency patterns using maximum covariance analysis [16]. Technically, the wavelet spectra are compared using a procedure based on the maximum covariance analysis that enables construction of a matrix of dissimilarities between wavelet power spectra over which a hierarchical clustering algorithm is applied. Then to quantify the synchrony in each subdivision, wavelet mean field (WMF) methodology was applied [17]. The magnitude of the WMF measures the strength of synchrony at both time period and frequency (periodic components). It is computed as a weighted average of normalized wavelet transforms of each time series [17]. Using WMF can characterize common periodic components and their potential time changes, then potential synchrony and its evolution. Significance levels of all different wavelet quantities were based on 1000 data-driven bootstrapped series with characteristics in agreement with those of the observed dataset [15].

## Results

### Spatio-temporal distribution of dengue cases

From August 1978 until October 2014 (435 months) the dengue database registered 48 261 geolocated results from laboratory analyses of which 14 382 were recorded as positive for DENV. Thirty-five islands (Windward– 3; Leeward– 5; Australs– 5; Marquesas– 6; Tuamotu-Gambier– 16) had at least one confirmed dengue case (S1 Table). The proportion of the population consulting for laboratory analyses from 1978 varied considerably among subdivisions, most notably being much lower in Tuamotu-Gambier (0.22%) compared to the other subdivisions (range 0.73% in the Australs to 1.31% in the Leeward islands). The age distribution of clinical presentations compared to the overall population was skewed by a higher percentage of children 0–1 years old presenting samples for testing (3.7% of this age group) and children aged 1–14 (1.7% of this age group) compared with 1% for older individuals. The age distribution of samples sent to the laboratory for analysis and those found positive was strongly correlated for both genders (Male r^2^ = 0.91; female r^2^ = 0.85; P<0.001).

**Fig 2:**
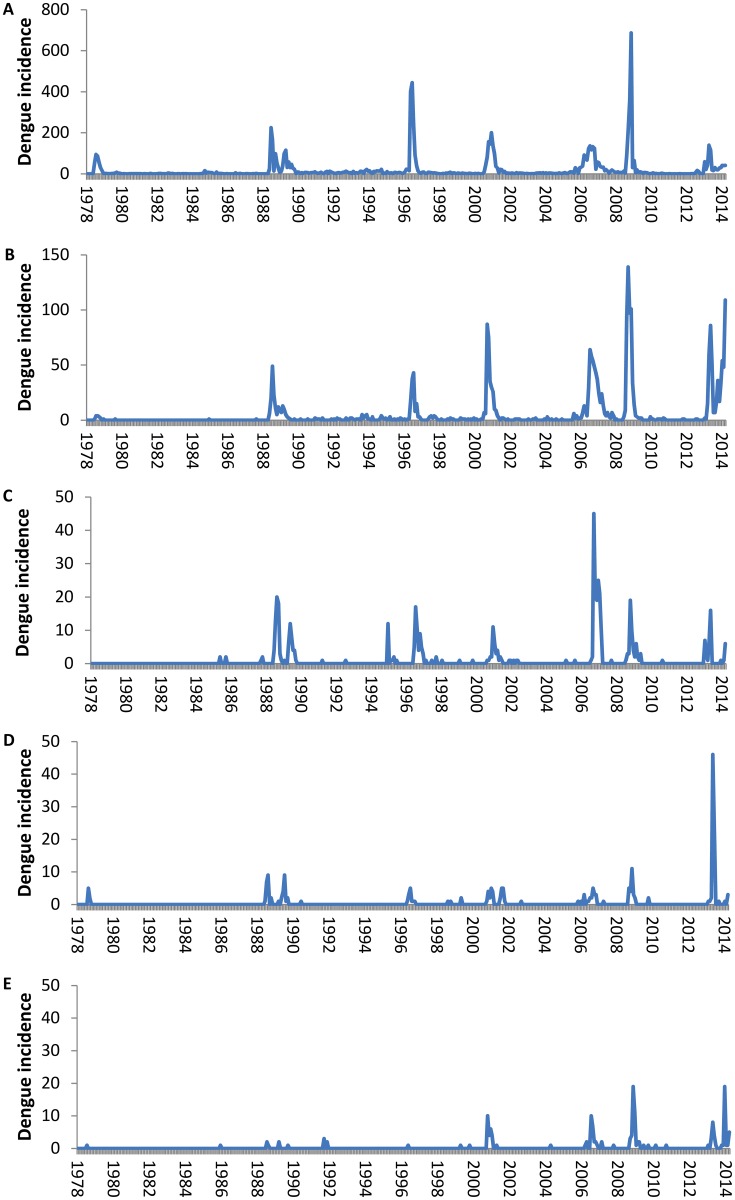
Incidence of confirmed dengue cases in French Polynesia from August 1978 until October 2014. Total number of dengue cases per month per subdivision of French Polynesia. A- Windward Islands, B. Leeward Islands, C. Marquesas Islands, D. Austral Islands, E. Tuamotu-Gambier Islands.

**Fig 3:**
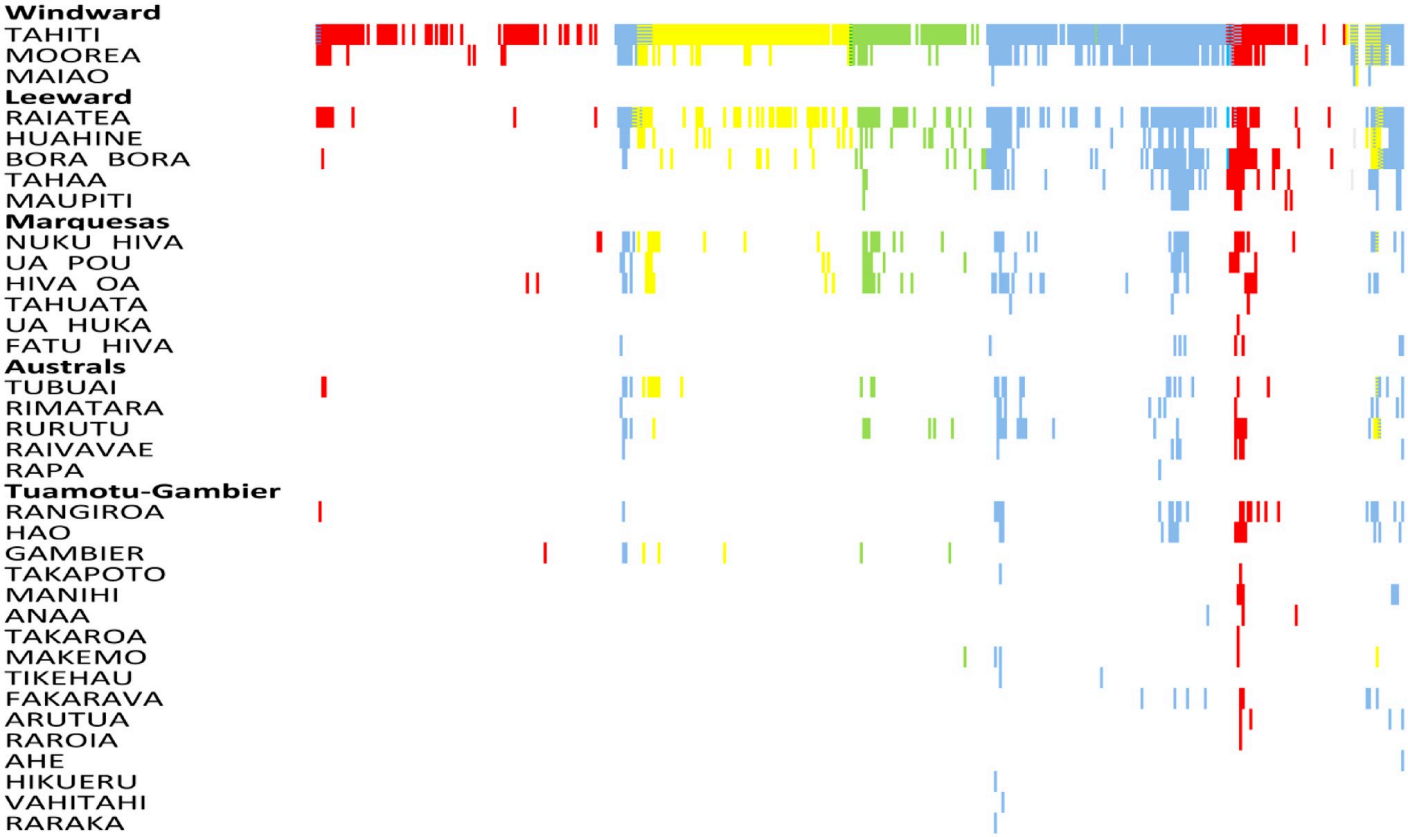
Occurrence of at least one dengue case per month per island. Serotypes: Blue: DENV-1; Green: DENV-2; Yellow: DENV-3; Red: DENV-4.

Over the 35-year period we defined eight epidemics (12 392 cases) and six inter-epidemic periods (1 990 cases) (Fig 2; S1 Text, S2 and S3 Tables). The corresponding occurrence of a dengue-incident month across all islands and according to DENV serotype is shown in Fig 3. Until 2013, DENV circulation was monotypic with very few occasions of co-circulation of more than one serotype at any one time. The majority of the co-circulation events occurred during the initial phases of the invasion of a novel serotype. Although islands from all subdivisions registered at least one incidence-month of dengue from the start of the 35-year period, an increasing number of islands reported cases over time, particularly in the less populated subdivisions, but which did not necessarily correspond to the amplitude of the dengue incidence. However, the overall number of cases was strongly positively correlated with the number of islands having at least one case during the month (Loglinear regression. Relative Risk (RR) per island number increase = 1.32, 95% Confidence intervals (CI) 1.30–1.34, P<0.001; S1 Fig).

**Fig 4:**
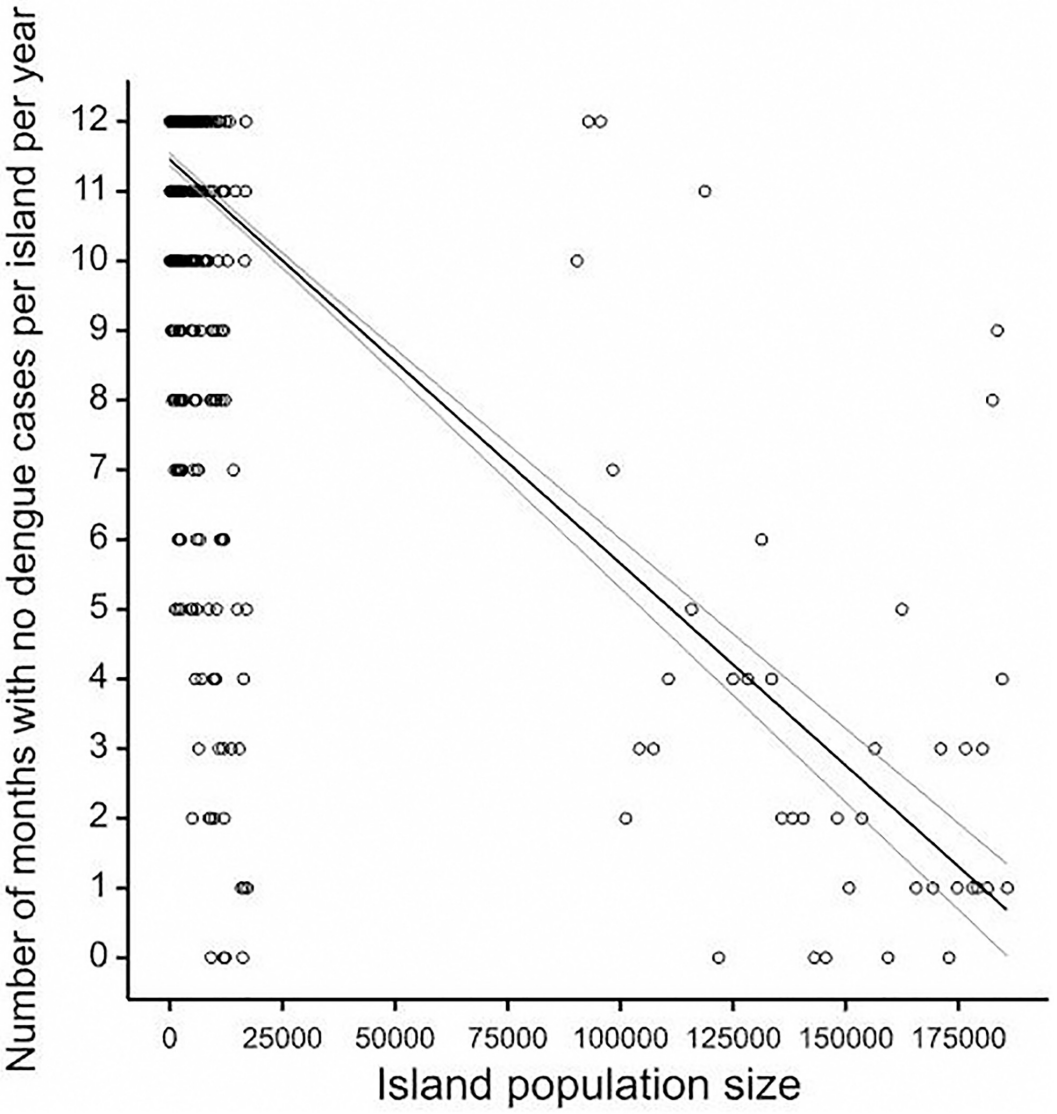
Critical community size of number of zero dengue case months per year per island.

Across the entire time period of 435 months, an absence of reported dengue cases for more than 1 month throughout French Polynesia was recorded on only 16 occasions (range 2–9 months), totalling 59 months (Fig 3). In Tahiti, the most populated island, there were no dengue cases for two or more consecutive months on only 17 occasions (range 2–14 months), totalling 76 months absence. From November 1988 until May 2011 dengue cases were observed at least every other month somewhere across French Polynesia. To assess the Critical community size (CCS) permitting continuous circulation of DENV, we plotted the number of months per year with no dengue per island and the corresponding island size. Classically, the CCS is taken as the point at which the regression line crosses the zero month absence point on the Y-axis. However, because DENV is mosquito-borne and can persist in the infected mosquito until it dies without being detectable in the human population, we relaxed the CCS definition to crossing the one month dengue absence per year Y axis point. The CCS is estimated to exceed 175 000 individuals (Linear regression. P<0.001; 40.9% variance explained) (Fig 4).

Island size being clearly a limiting factor for DENV persistence, we then addressed two population size dependent phenomena: initial viral establishment in an island population and subsequent duration of viral transmission within the island.

The probability of DENV establishing itself for at least two consecutive dengue incident months was assessed as a function of island population size, the number of cases in the first month and DENV serotype. We first assumed that at least two consecutive months with dengue cases was evidence of within-island transmission and not simply importation. The probability of consecutive dengue incident months increased with both population size and number of cases in Month 1, reaching an asymptote at a population size in excess of 10 000 individuals and 30 dengue cases (Multivariate logistic regression, Log(10) population size: Odds Ratio (OR) for unit increase in log (10) population size = 3.36, 95% CI 2.68–4.22, P<0.001; Log(10) cases: OR for unit increase in log (10) case number = 21.7, 95% CI 13.6–34.4, P<0.001) (S2 and S3 Figs). This suggests that the number of cases in month 1 has a strong impact on the probability of subsequent incident months up to a relatively low number of cases, beyond which there is no increased probability. This increased risk is also observable with population size, but to a lesser extent (lower OR). DENV serotype (DENV-1, -2, -3, -4, or mixed -1 and -3) was not found to be associated with the probability of DENV transmission during two consecutive months (Wald χ^2^_4_ = 7.62, P = 0.17). Re-analysis excluding Tahiti revealed the same asymptote for dengue cases but no asymptote whatever the population size, with increase in probability up to the maximum population size of approximately 17 000 in Moorea. Increasing the duration requirement to three consecutive months across all islands yielded an increased OR for log (10) population size (OR = 4.76, 95%CI 3.80–5.96) and for log (10) cases (OR = 36.56, 95%CI 21.35–62.65). No population size asymptote was attained, but again an asymptote in the probability of having three consecutive months of transmission was observed at approximately 30 cases in the first month.

We next addressed the duration of viral transmission following at least two consecutive dengue incident months (when local viral transmission was presumed to be occurring) within an island as a function of population size, cumulative number of cases and DENV serotype. Local transmission was presumed to have stopped when there were three consecutive zero case months. The duration of viral circulation until three consecutive zero case months increased with island population size (RR = 1.7, 95%CI 1.39–2.07, P<0.001) and the cumulative number of dengue cases (RR = 2.49, 95%CI 2.13–2.91, P<0.001) (S4 and S5 Figs). DENV circulation was also longer for DENV-1 as compared to DENV-2, DENV-4 and the mixed DENV-1/3 epidemic (DENV-2: RR = 0.71, 95%CI 0.53–0.96, P = 0.025; DENV-4: RR = 0.63, 95%CI 0.51–0.79, P<0.001; DENV-1/3: RR = 0.59, 95%CI 0.41–0.85, P = 0.004), but not DENV-3 (RR = 0.77, 95%CI 0.57–1.03, P = 0.076) (S4 and S5 Figs).

### Incidence synchrony among subdivisions and islands

**Fig 5:**
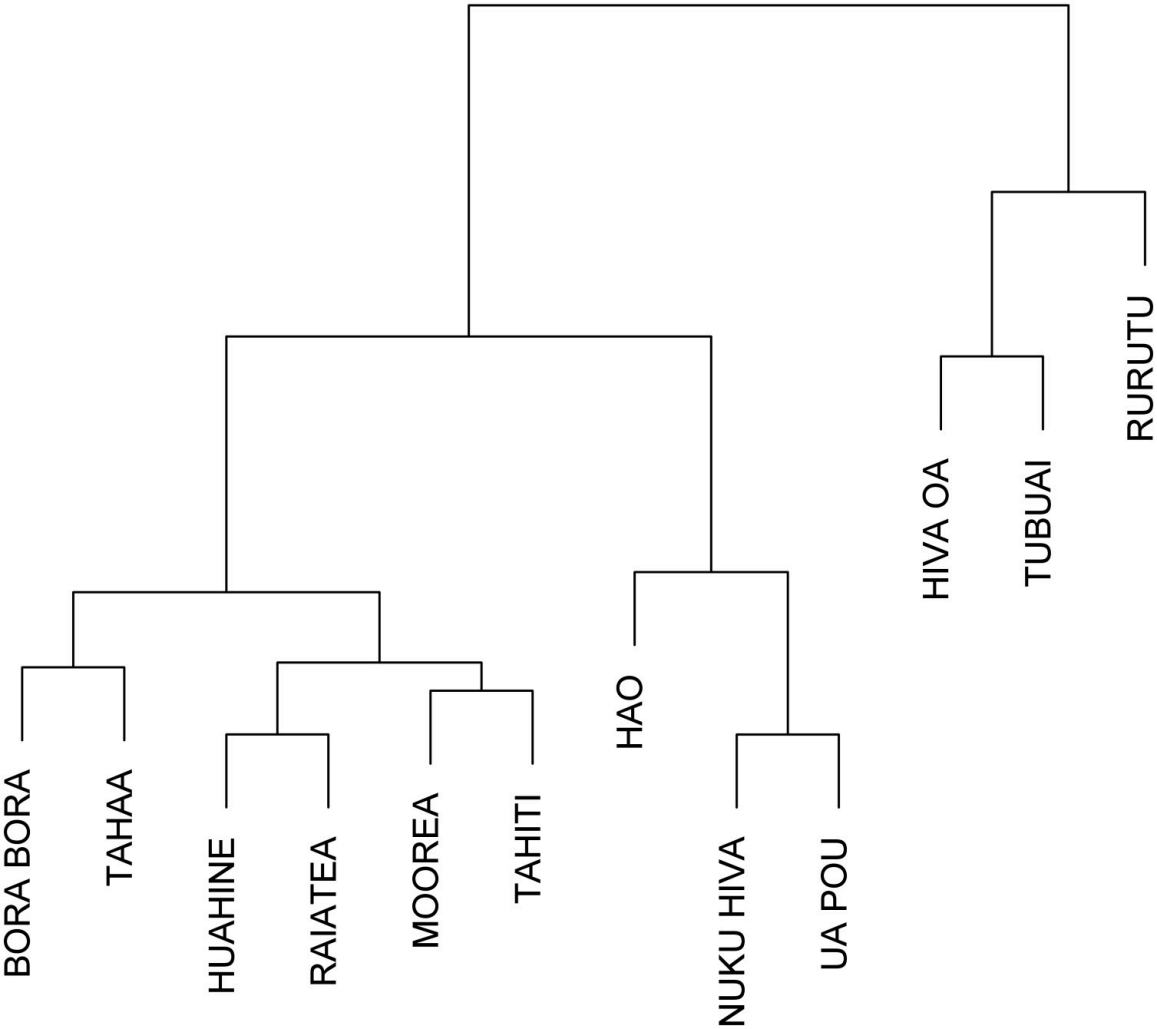
Dendrogram of wavelet clustering showing clusters with similar spectral features.

**Fig 6:**
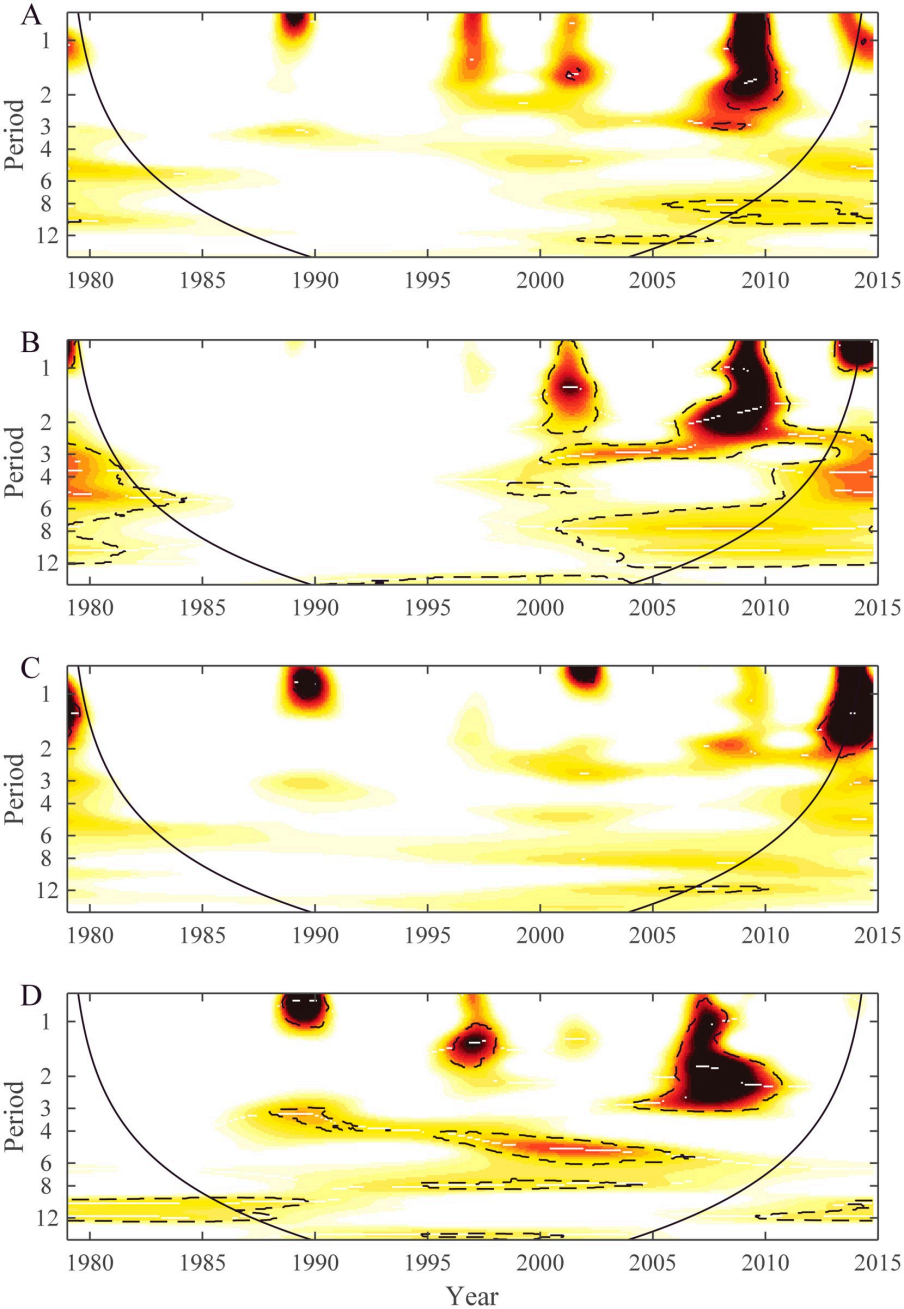
Wavelet mean field (WMF) spectra of dengue incidence time series for four subdivisions: A. Leeward, B. Windward, C. Australs and D. Marquesas. The plots show WMF representing common variance structures; common variance and synchrony (dark red) and absence of common variance (white). The dashed lines denote the 5% significance areas determined with a bootstrapping scheme based on a Hidden Markov process [15]. The black line defines the cone of influence below which the information is affected by edge effect.

Wavelet power spectrum of time series data from 12 of the most populated islands of French Polynesia are displayed in S6 and S7 Figs. These 12 time series were chosen because they had enough dengue cases. In general, periodicities were detected in the 1-year, 2-year, 2–3-year and 6-year bands emphasizing that the seasonality is not marked and that each epidemic wave can last longer than 1 year. However, dengue dynamics varied across the 12 time series. To analyze these differences between time series wavelet cluster analysis [16] was performed and showed coherent dynamics within each subdivision. Fig 5 shows the dendrogram obtained from wavelet clustering. Except for the time series from Hiva Oa, all others time series group by subdivision. Taking into account this subdivision arrangement for dengue dynamics, we applied WMF to quantify the synchrony inside each subdivision. Fig 6 displays the results from WMF showing, for each subdivision, the common significant periodic components for which time series are synchronized highlighting each epidemic wave. This analysis demonstrates the differences among subdivisions in dengue periodicity, notably over the 1-year, 2-year, 2–3-year and 6-year periods. However, it is notable that the 2009–2010 DENV-4 epidemic is synchronous in three subdivisions (Leeward, Windward and Marquesas islands), with a significant periodicity spreading over the 1–3 year band highlighting again the absence of seasonality of this epidemic.

We then performed cross-correlation analyses firstly for all islands within each subdivision with the “capital” island of the subdivision and then all islands with Tahiti. Capital islands of each subdivision are defined as those being both highly inhabited and most connected to the other islands by air and boat. Individuals can island hop but their specific place of departure is not known, thus islands may be indirectly connected to Tahiti (or other capital islands). There was a significant cross-correlation with zero month lag for nine islands. Five islands showed no significant temporal association, three showed association with a positive lag (later than Tahiti) and three islands had a negative lag association. Notably Bora Bora and Tahaa precede Tahiti by two months. Cross-correlations within each of the other four subdivisions using the major island of each subdivision as the lead island yielded improved correlation (increased χ^2^). Overall, when significant, most cross-correlations were either at zero lag or negative lag, suggesting that the smaller islands are synchronised with or leading the”capital” island within each archipelago (Table 1).

**Table 1:** Cross-correlation over entire 435 month period of monthly incidence of dengue in all islands with Tahiti and with capital islands within each subdivision. Post Bonferroni correction non-significantly correlated islands in italics.

	Zero lag	Negative lag		Positive lag		No cross
Island	Island	Island	Month lag	Island	Month lag	correlation
**Tahiti** (Windward)	Gambier	Bora Bora	-2	Nuku Hiva	1	Manihi
	*Hao*	Tahaa	-2	*Hiva Oa*	1	Fakarava
	*Rangiroa*	Rimatara	-2	Tubuai	1	Fatu Hiva
	Ua Pou					Maiao
	Huahine					Rurutu
	Raiatea					
	Maupiti					
	Moorea					
	Raivavae					
**Raiatea** (Leeward)	Huahine	Bora Bora	-1			
	Maupiti	Tahaa	-2			
**Nuku Hiva** (Marquesas)		Fatu Hiva	-3	Hiva Oa	8	
		Ua Pou	-2			
		Hiva Oa	-1			
**Tubuai** (Australs)		Raivavae	-1			Rurutu
						Rimatara
**Rangiroa** (Tuamotu)	Hao					Gambier
	*Fakarava*					Manihi

**Fig 7:**
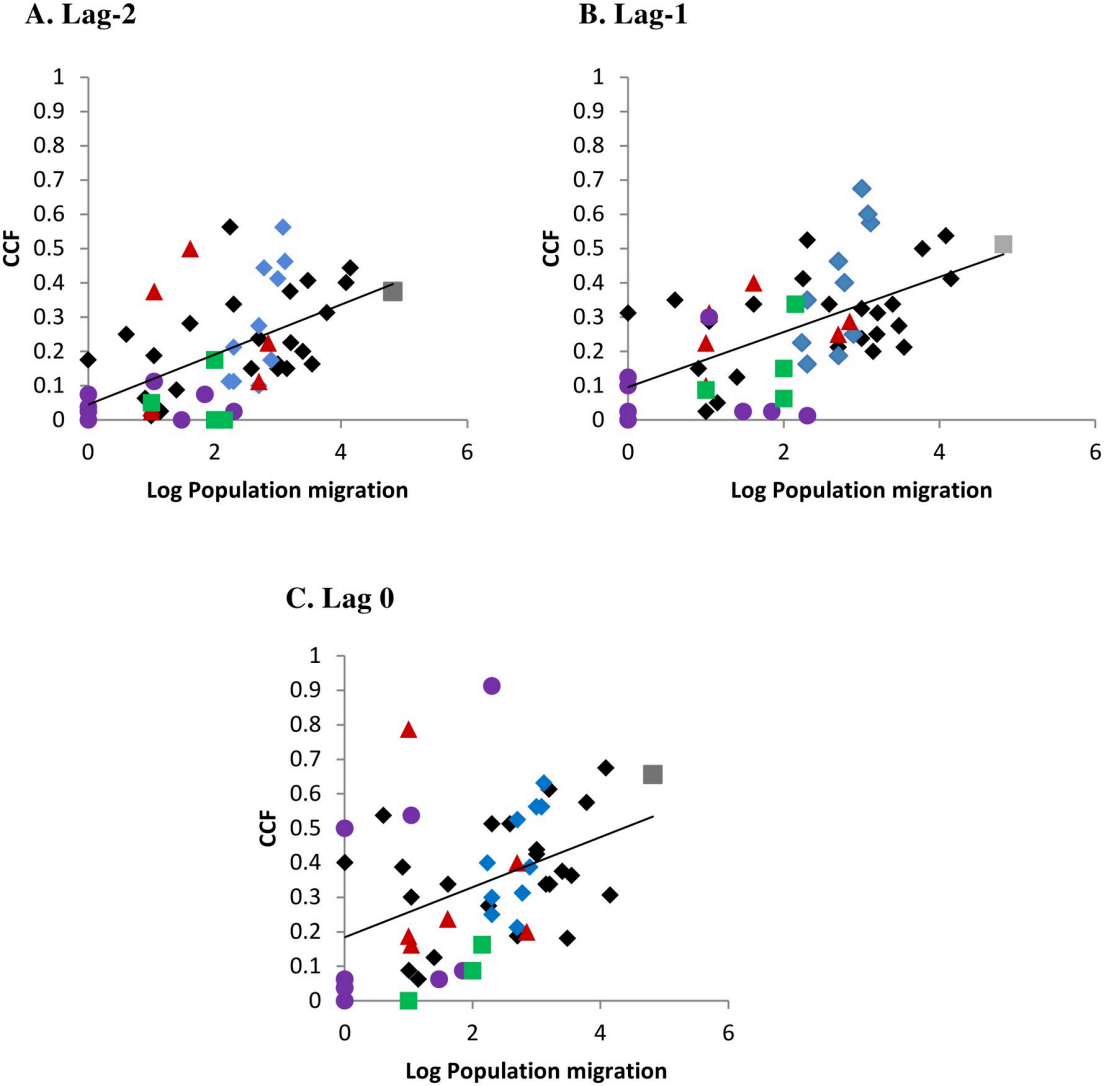
Cross-correlation of dengue incidence rate among connected islands with known extent of population migration (log10 transformed). Given are the cross-correlation functions (CCF) at different lag months between the lead and following island. Colours represent within subdivision island correlations: Grey square—Windward Islands, Blue diamonds—Leeward Islands, Brown triangles—Marquesas Islands, Green squares—Austral Islands, Mauve circles—Tuamotu-Gambier Islands. Black colour indicates among subdivision island correlations. Lines of tendency: Lag -2 month r^2^ = 0.29; Lag -1 month r^2^ = 0.32; Lag 0 month r^2^ = 0.16. Population migration data used was air travel in 2010 (S4 Table).

As shown in Fig 7, there was an increase in the correlation of monthly incidence rate with increasing population exchange, using air travel data from 2010 (S4 Table). As indicated by different colours representing island correlations within subdivision (and black being among subdivision island correlations), the relationship was not influenced by subdivision *per se* but the extent of population exchange. Furthermore, the correlation (of CCF with population movement) was greater at months lag-1, lag-2 than at lag 0; this highlights the overall lack of immediate synchrony of the dengue incidence rate across connected populations.

## Discussion

During the 35-year study period, dengue epidemiology was characterized by intermittent mono-serotype epidemics followed by inter-epidemic low circulation periods. With exception of Tahiti, the most populated island of French Polynesia, viral die-out at an island level was frequent and the majority of the less populated islands had few cases and rarely for more than 2 consecutive months. Indeed, according to our analyses, there was a reduced probability of islands smaller than 10 000 individuals having even two consecutive dengue positive months and a strong impact of population size on the duration of viral circulation once initiated. This would suggest that the virus is unable to take hold in these conditions, whether due to previously acquired immunity or human or mosquito population density [18, 19]. Despite this, almost permanent circulation of virus was observed for decades across French Polynesia and the estimated CCS of 175 000 was only attained in Tahiti in 2002 [10]. Implicit in this study was an assessment of whether there is evidence that a metapopulation approach to understanding the dynamics of dengue in French Polynesia is useful, based on classical concepts of synchrony and persistence. Dengue incidence was largely asynchronous across subdivisions, but partially synchronous within subdivision with correlations in dengue incidence occurring at zero or small lag times. It was also notable that correlations in incidence increased with inter-island population migration, irrespective of sub-division. These observations would support a meta-population structure to transmission within French Polynesia, where the virus hops from island to island, especially given that the critical community size to maintain virus was only achieved in Tahiti in 2002. However, the contribution of inapparent, subclinical infections cannot be underestimated and may be sufficient for Tahiti alone to maintain a source of transmission [8].

More generally across the entirety of French Polynesia, three of the eight epidemics fizzled out after a relatively short period without any apparent change in climatic conditions and the virus continued circulating at a low level for an extended time before finally dying out. These three epidemics are: the DENV-4 1979 epidemic that lasted for 6 months but then persisted at a low level for another 113 months; the DENV-2 1996/7 epidemic that lasted for 9 months, before persisting for another 42 months; and the DENV-4 2009 epidemic that lasted for 8 months, before persisting for additional 15 months at sub-epidemic levels (S2 Table). However, it is unclear whether DENV-2 died out alone as the last DENV-2 case was reported in December 2000 and the first DENV-1 case was reported the following month. Interestingly, two of the situations, where self-extinction occurred several months before the detection of a new serotype, involved DENV-4.

By contrast, in the other epidemics, there is clear suggestion that invasion of a new serotype excludes the current serotype. Within serotype competitive replacement among clades has been seen in many studies [20], where viral diversification during epidemics subsequently leads to purifying selection through immune selection in humans and mosquitoes [21]. However, serotype replacement through competition has rarely been observed in most endemic natural settings [22], but seems to be a feature of the South Pacific region [23–26], except for French Polynesia.

Epidemic recrudescence of a persisting DENV occurred in 2006, with the same viral strain as in 2001 [27]. Judging from the time series (Fig 2), it would seem that the second epidemic must have occurred following expansion in the Windward Islands with subsequent exportation to the Marquesas that had been spared the 2001 epidemic.

How in French Polynesia, whatever the serotype, DENV circulation can persist between epidemics for several months with only few confirmed cases is perplexing. Viral maintenance by vertical transmission (VT) in mosquitoes might have occurred [28], but the role of VT is equivocal and the climatic conditions should have enabled active transmission. A significant role of inapparent infections that then led to viral transmission within naïve sub-populations seems a likely explanation.

Analyses using only laboratory confirmed cases underestimates the true burden of infection in the population where the majority are likely to be sub-clinical [8, 29] and will be additionally confounded by both the vagaries of doctors’ demands for analyses to be performed and unequal access to health care facilities depending on the subdivision. This is especially evident in the Tuamotu-Gambier subdivision, from where there were few analyses performed and low confirmed dengue rates, but which was subsequently shown to have the same sero-prevalence rates as the other subdivisions [30]. An additional confounding factor in this study was that increasingly sensitive diagnostic techniques were used over time, thus making comparisons among epidemics untenable. However, insofar as this work analysed the data, for the most part, within time-period and across islands, temporal variation in diagnostic sensitivity should not alter our conclusions significantly. One feature not considered in this present study was the association of dengue incidence with climate. In French Polynesia, climate is suitable for dengue transmission year round with little variation in ambient temperature and the epidemics observed did not coincide with any major El Niño-Southern Oscillation events. However, the different nature of the islands, high islands as compared to atolls, could generate variability in local climate, especially rainfall and hence suitability for dengue transmission. Such island specific variation could differentially impact the different mosquito species that occur in the larger islands (*Aedes aegypti*) compared to the smaller outer islands (*Aedes polynesiensis*)[31,32]. Both are capable of transmitting virus but have different ecologies and thus may be differentially affected by variation in climatic factors. With suitable environmental data at an island level, if available, further analysis would be of interest.

Spatial structuring of dengue epidemiology has been reported at micro- [33–37], meso- [6, 7, 37–39] and macro-scales [39–44]. At increasing scales, human mobility becomes the major vector of virus, potentially providing the source of infection to generate novel, distal clusters of dengue [37, 45]. This seems likely to be occurring to some extent in French Polynesia, but limited to the most inhabited islands. Inter-connectedness of islands does seem to enable dispersal of the virus throughout the territory, but with low probability of a subsequent epidemic occurring in the outlying islands. The long-term persistence of DENV during inter-epidemic periods is remarkable and suggests that the within-island scale needs to be addressed as the foci of infection are likely to be very spatially limited. The impact of inapparent infections is likely to be non-negligible and sero-surveys across the subdivisions would reveal the extent to which herd immunity rather than stochastic viral die-out tempers the progression of epidemic viral transmission.

## Supporting information

S1 Text Epidemic and inter-epidemic period definition. (DOCX)

S1 Fig Relationship between the number of islands with dengue cases and the sum of the monthly dengue cases across all French Polynesia. Shown is the best fit line from the GLM loglinear regression.
(TIF)

S2 Fig Probability of having a second consecutive month positive for a dengue case as a function of Island Log(10) population size. Shown are the best fit lines from the GLM logistic regression and the adjusted probabilities (partial residuals) from the multivariate analysis.
(TIF)

S3 Fig Probability of having a second consecutive month positive for a dengue case as a function of Log(10) number of cases in the first month. Shown are the best fit lines from the GLM logistic regression and the adjusted probabilities (partial residuals) from the multivariate analysis.
(TIF)

S4 Fig Duration of viral transmission per island as a function of Island Log(10) population size and serotype. Shown are the best fit lines from the GLM loglinear regression and the adjusted probabilities (partial residuals) from the multivariate analysis. Serotype colour codes are: DENV-1 red; DENV-2 green; DENV-3 blue; DENV-4 cyan; DENV1+3 pink.
(TIF)

S5 Fig Duration of viral transmission per island as a function of Log(10) cumulative number of cases including first two months of dengue case occurrence and serotype. Shown are the best fit lines from the GLM loglinear regression and the adjusted probabilities (partial residuals) from the multivariate analysis. Serotype colour codes are: DENV-1 red; DENV-2 green; DENV-3 blue; DENV-4 cyan; DENV1+3 pink.
(TIF)

S6 Fig Wavelet power spectrum of time series data from the first 6 of the 12 major islands of French Polynesia. Left panel: Local wavelet power spectrum. Colours code for power values from white (low values) to dark red (high values). The black line defines the cone of influence below which the information is affected by edge effect. White lines represent the maxima of the undulations of the wavelet power spectrum. Right panel: Average wavelet power spectrum. For both panels, the dashed lines denote the 5% significance areas determined with a bootstrapping scheme based on a Hidden Markov process [14]. A: Bora Bora; B: Hao; C: Hiva Oa; D: Huahine; E: Moorea; F: Nuku Hiva.
(TIF)

S7 Fig Wavelet power spectrum of time series data from the second 6 of the 12 major islands of French Polynesia. Left panel: Local wavelet power spectrum. Colours code for power values from white (low values) to dark red (high values). The black line defines the cone of influence below which the information is affected by edge effect. White lines represent the maxima of the undulations of the wavelet power spectrum. Right panel: Average wavelet power spectrum. For both panels, the dashed lines denote the 5% significance areas determined with a bootstrapping scheme based on a Hidden Markov process [14]. G: Raiatea; H: Rurutu; I: Tahaa; J: Tahiti; K: Tubuai; L: Ua Pou.
(TIF)

S1 Table Dengue circulation within islands. Population size is the mid-term value from the censuses [10]. Bold type for island showing the highest number of incidence months within each subdivision. Colour coding for different subdivisions.
(DOCX)

S2 Table Summary data of the 8 epidemic and 6 inter-epidemic periods of dengue transmission between August 1978 and October 2014. Given are the etiological serotype and genotype, the start date and duration, the number of subdivisions and islands affected and the total number of samples tested and found positive for DENV. NK–not known.
(DOCX)

S3 Table Number of confirmed dengue cases by subdivision and epidemic and inter-epidemic period between August 1978 and October 2014. (DOCX)

S4 Table Air travel between islands in 2010. (DOCX)
