# Supplementary material for: Long-term persistence of monotypic dengue transmission in small size isolated populations, French Polynesia, 1978-2014
Source: PLoS Negl Trop Dis. 2020 Mar 6;14(3):e0008110. doi: 10.1371/journal.pntd.0008110 (PMC7080275; doi:10.1371/journal.pntd.0008110)
Supplement: S1 Text — (DOCX) [file pntd.0008110.s001.docx]

**Text S1. Epidemic and Inter-Epidemic Period definition**

Over the study period, French Polynesia Public Health authorities defined a dengue epidemic using an arbitrary incidence of 10 cases per week. To create a more objective threshold, dengue epidemic periods were here defined as starting when the incidence rate (IR) within any of the five subdivisions was above the background threshold of that subdivision for two consecutive months and ending when the IR was below the threshold for two consecutive months. The background threshold was calculated for each subdivision separately as follows: dengue die-out within a subdivision was considered to have occurred when there were no dengue cases for three consecutive months, and thus all consecutive months with zero cases and equal to or greater than three months were removed from the background threshold calculation. We used Tukey’s box-plot method to identify outliers: the inter-quartile (IQ) range of monthly case numbers was calculated and the outlier threshold was defined as the 3rd quartile plus 1.5 x the IQ range. Any month when this value occurred or was surpassed (denoted outliers) was then removed and the procedure repeated until there were no outliers, yielding the background threshold of monthly incidence.

Using our threshold values, from 1978 until 2014, a total of seven epidemic periods were identified, one of which (1988-Dec to 1990-May) could be further sub-divided into a twin epidemic (Epi 2 and 3) due to the invasion of a novel serotype (S2 Table).
