# Supplementary figures and images for: Long-term persistence of monotypic dengue transmission in small size isolated populations, French Polynesia, 1978-2014
Source: PLoS Negl Trop Dis. 2020 Mar 6;14(3):e0008110. doi: 10.1371/journal.pntd.0008110 (PMC7080275; doi:10.1371/journal.pntd.0008110)

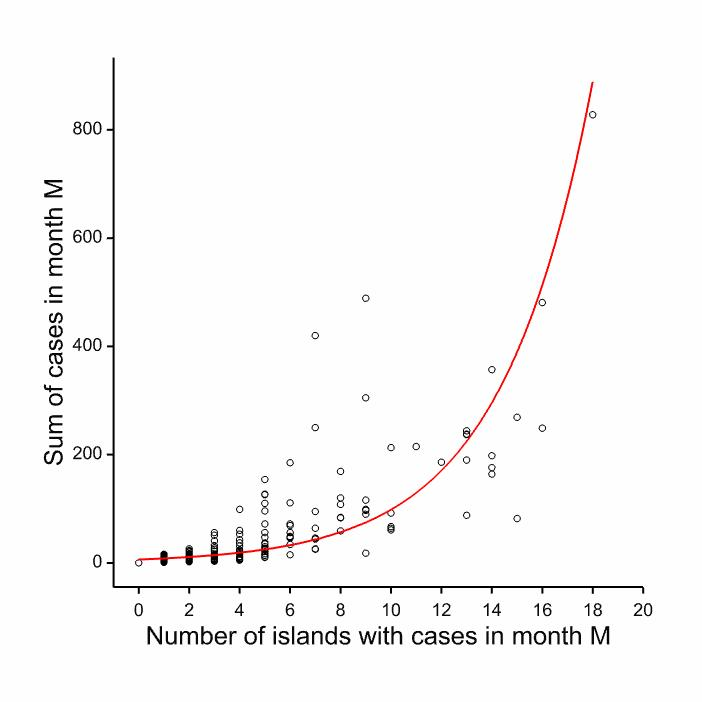

Supplement: S1 Fig — Shown is the best fit line from the GLM loglinear regression. (TIF) [file pntd.0008110.s002.tif]

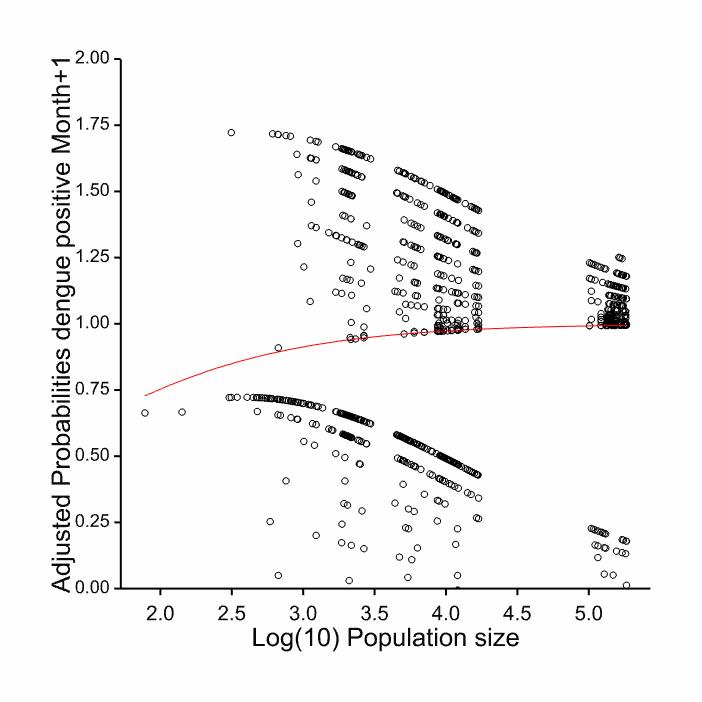

Supplement: S2 Fig — Shown are the best fit lines from the GLM logistic regression and the adjusted probabilities (partial residuals) from the multivariate analysis. (TIF) [file pntd.0008110.s003.tif]

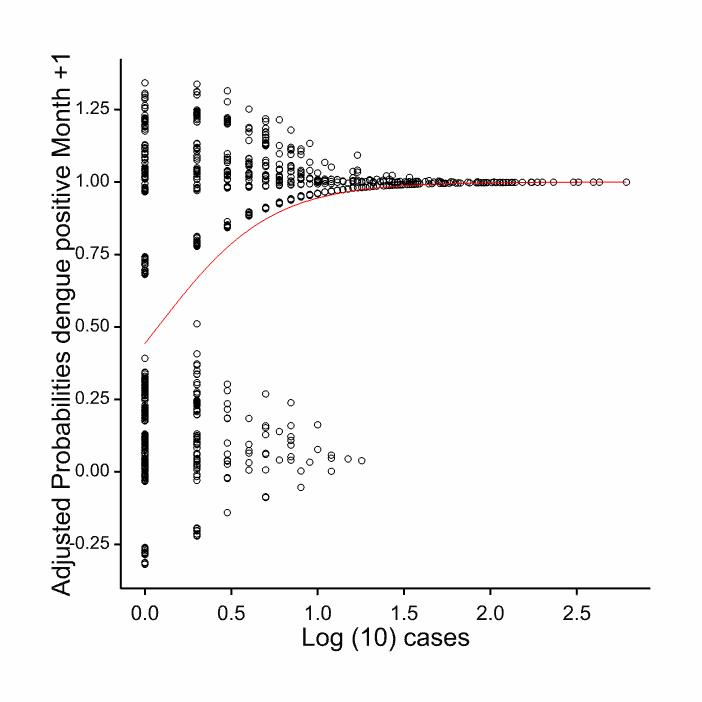

Supplement: S3 Fig — Shown are the best fit lines from the GLM logistic regression and the adjusted probabilities (partial residuals) from the multivariate analysis. (TIF) [file pntd.0008110.s004.tif]

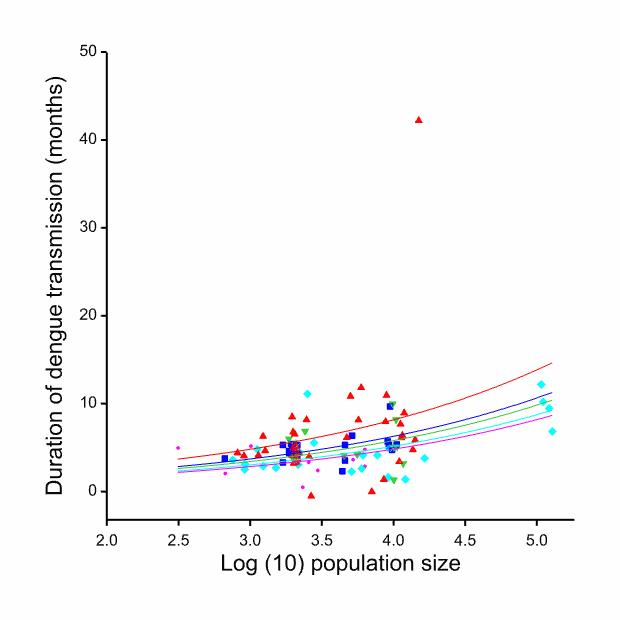

Supplement: S4 Fig — Shown are the best fit lines from the GLM loglinear regression and the adjusted probabilities (partial residuals) from the multivariate analysis. Serotype colour codes are: DENV-1 red; DENV-2 green; DENV-3 blue; DENV-4 cyan; DENV1+3 pink. (TIF) [file pntd.0008110.s005.tif]

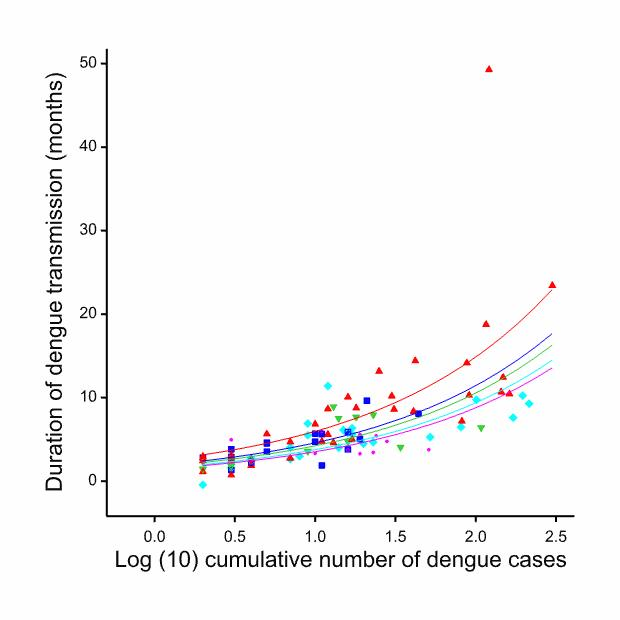

Supplement: S5 Fig — Shown are the best fit lines from the GLM loglinear regression and the adjusted probabilities (partial residuals) from the multivariate analysis. Serotype colour codes are: DENV-1 red; DENV-2 green; DENV-3 blue; DENV-4 cyan; DENV1+3 pink. (TIF) [file pntd.0008110.s006.tif]

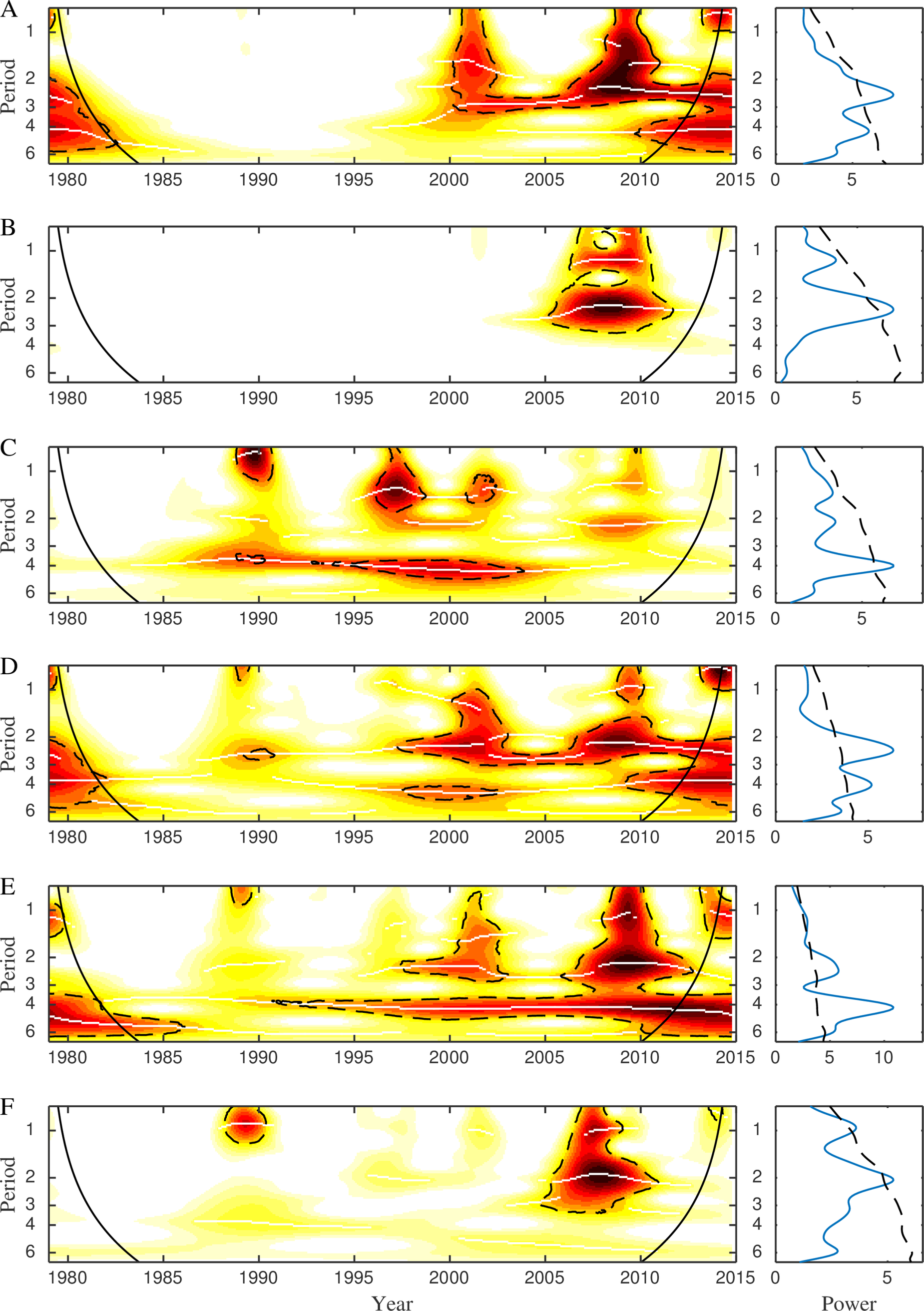

Supplement: S6 Fig — Left panel: Local wavelet power spectrum. Colours code for power values from white (low values) to dark red (high values). The black line defines the cone of influence below which the information is affected by edge effect. White lines represent the maxima of the undulations of the wavelet power spectrum. Right panel: Average wavelet power spectrum. For both panels, the dashed lines denote the 5% significance areas determined with a bootstrapping scheme based on a Hidden Markov process [14]. A: Bora Bora; B: Hao; C: Hiva Oa; D: Huahine; E: Moorea; F: Nuku Hiva. (TIF) [file pntd.0008110.s007.tif]

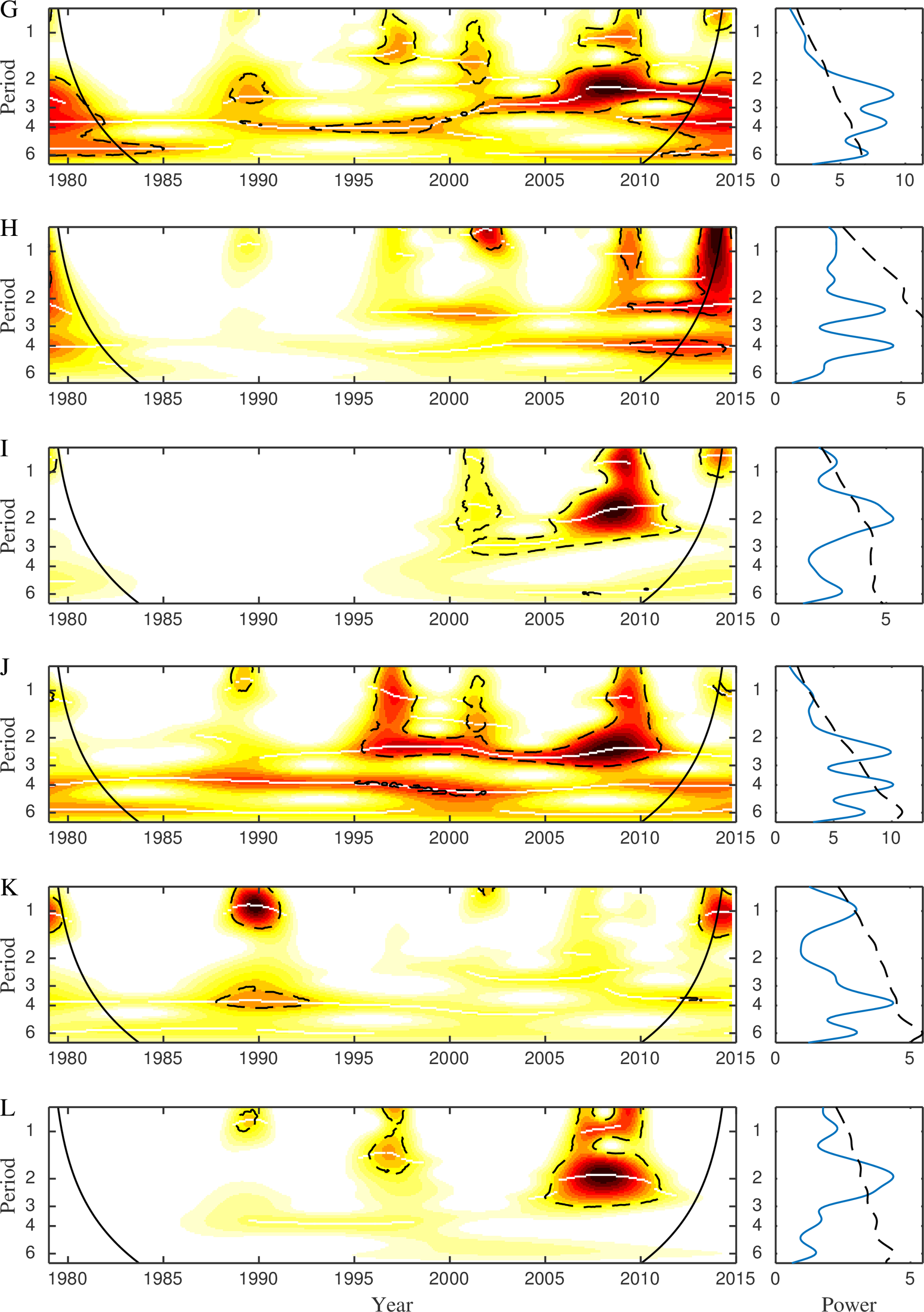

Supplement: S7 Fig — Left panel: Local wavelet power spectrum. Colours code for power values from white (low values) to dark red (high values). The black line defines the cone of influence below which the information is affected by edge effect. White lines represent the maxima of the undulations of the wavelet power spectrum. Right panel: Average wavelet power spectrum. For both panels, the dashed lines denote the 5% significance areas determined with a bootstrapping scheme based on a Hidden Markov process [14]. G: Raiatea; H: Rurutu; I: Tahaa; J: Tahiti; K: Tubuai; L: Ua Pou. (TIF) [file pntd.0008110.s008.tif]
