# Supplementary material for: Long-term persistence of monotypic dengue transmission in small size isolated populations, French Polynesia, 1978-2014
Source: PLoS Negl Trop Dis. 2020 Mar 6;14(3):e0008110. doi: 10.1371/journal.pntd.0008110 (PMC7080275; doi:10.1371/journal.pntd.0008110)
Supplement: S1 Table — Population size is the mid-term value from the censuses [10]. Bold type for island showing the highest number of incidence months within each subdivision. Colour coding for different subdivisions. (DOCX) [file pntd.0008110.s009.docx]

**Table S1**  Dengue circulation within islands. Population size is the mid-term value from the censuses [10]. Bold type for island showing the highest number of incidence months within each subdivision. Colour coding for different subdivisions.

| Island | Population size | Subdivision | Number of incidence months | Total months since first record | Year-month first incidence | Month start |
| --- | --- | --- | --- | --- | --- | --- |
| **TUBUAI** | **1846** | **Aust** | **33** | **428** | **1979-3** | **8** |
| RURUTU | 1953 | Aust | 30 | 309 | 1989-2 | 127 |
| RAIVAVAE | 1225 | Aust | 9 | 309 | 1989-2 | 127 |
| RIMATARA | 969 | Aust | 11 | 310 | 1989-1 | 126 |
| RAPA | 516 | Aust | 1 | 98 | 2006-10 | 338 |
| **TAHITI** | **131309** | **Win.** | **360** | **435** | **1978-8** | **1** |
| MOOREA | 8801 | Win. | 138 | 430 | 1979-1 | 6 |
| MAIAO | 231 | Win. | 6 | 163 | 2001-4 | 273 |
| **RAIATEA** | **8560** | **Lee** | **155** | **430** | **1979-1** | **6** |
| HUAHINE | 4479 | Lee | 65 | 310 | 1989-1 | 126 |
| BORA.BORA | 4225 | Lee | 82 | 428 | 1979-3 | 8 |
| TAHAA | 4005 | Lee | 44 | 214 | 1997-1 | 222 |
| MAUPITI | 963 | Lee | 16 | 214 | 1997-1 | 222 |
| **NUKU.HIVA** | **2100** | **Marq** | **47** | **319** | **1988-4** | **117** |
| UA.POU | 1918 | Marq | 28 | 310 | 1989-1 | 126 |
| HIVA.OA | 1671 | Marq | 40 | 347 | 1985-12 | 89 |
| TAHUATA | 633 | Marq | 3 | 156 | 2001-11 | 280 |
| UA.HUKA | 539 | Marq | 1 | 66 | 2009-5 | 370 |
| FATU.HIVA | 497 | Marq | 9 | 310 | 1989-1 | 126 |
| **RANGIROA** | **1305** | **Tuamo** | **26** | **429** | **1979-2** | **7** |
| HAO | 1156 | Tuamo | 13 | 160 | 2001-7 | 276 |
| GAMBIER | 620 | Tuamo | 15 | 340 | 1986-7 | 96 |
| TAKAPOTO | 465 | Tuamo | 2 | 160 | 2001-7 | 276 |
| MANIHI | 429 | Tuamo | 6 | 66 | 2009-5 | 370 |
| ANAA | 426 | Tuamo | 3 | 78 | 2008-5 | 358 |
| TAKAROA | 396 | Tuamo | 1 | 66 | 2009-5 | 370 |
| MAKEMO | 373 | Tuamo | 4 | 174 | 2000-5 | 262 |
| TIKEHAU | 312 | Tuamo | 2 | 160 | 2001-7 | 276 |
| ARUTUA | 288 | Tuamo | 3 | 65 | 2009-6 | 371 |
| FAKARAVA | 248 | Tuamo | 9 | 214 | 1997-1 | 222 |
| RAROIA | 166 | Tuamo | 1 | 65 | 2009-6 | 371 |
| AHE | 162 | Tuamo | 1 | 1 | 2014-10 | 435 |
| HIKUERU | 123 | Tuamo | 1 | 162 | 2001-5 | 274 |
| VAHITAHI | 76 | Tuamo | 1 | 159 | 2001-8 | 277 |
| RARAKA | 63 | Tuamo | 1 | 162 | 2001-5 | 274 |
