# Supplementary material for: Long-term persistence of monotypic dengue transmission in small size isolated populations, French Polynesia, 1978-2014
Source: PLoS Negl Trop Dis. 2020 Mar 6;14(3):e0008110. doi: 10.1371/journal.pntd.0008110 (PMC7080275; doi:10.1371/journal.pntd.0008110)
Supplement: S2 Table — Given are the etiological serotype and genotype, the start date and duration, the number of subdivisions and islands affected and the total number of samples tested and found positive for DENV. NK–not known. (DOCX) [file pntd.0008110.s010.docx]

**Table S2** Summary data of the 8 epidemic and 6 inter-epidemic periods of dengue transmission between August 1978 and October 2014 giving the etiological serotype and genotype, the start date and duration, the number of subdivisions and islands affected and the total number of samples tested and found positive for DENV. NK – not known.

|  |  |  | Start date | Duration | N° | N° | Samples |  | Serotype of +ve samples | | | | |
| --- | --- | --- | --- | --- | --- | --- | --- | --- | --- | --- | --- | --- | --- |
| Period | Serotype | Genotype | /place | (months) | Subdivisions | Islands | tested | DENV+ | NK | 1 | 2 | 3 | 4 |
| Epi1 | 4 | 2b | 1979-1 / Wind | 6 | 4 | 6 | 863 | 444 | 276 | 4 | 0 | 0 | 164 |
| InterEpi |  |  |  | 113 | 4 | 6 | 2829 | 329 | 201 | 0 | 1 | 0 | 130 |
| Epi2 | 1 | V | 1988-12 / Wind | 8 | 5 | 15 | 4010 | 1635 | 624 | 991 | 0 | 20 | 0 |
| Epi3 | 3 | I | 1989-8 / Lee-Wind | 11 | 5 | 11 | 5028 | 1326 | 515 | 59 | 0 | 753 | 0 |
| InterEpi |  |  |  | 76 | 4 | 9 | 6493 | 1017 | 424 | 0 | 28 | 565 | 0 |
| Epi4 | 2 | Cosmopolitan | 1996-11 / Wind | 9 | 5 | 13 | 3127 | 1735 | 1077 | 0 | 657 | 1 | 0 |
| InterEpi |  |  |  | 42 | 5 | 12 | 1310 | 186 | 185 | 0 | 1 | 0 | 0 |
| Epi5 | 1 | IV | 2001-2 / Lee | 16 | 5 | 25 | 3887 | 1541 | 1456 | 85 | 0 | 0 | 0 |
| InterEpi |  |  |  | 44 | 4 | 9 | 1433 | 182 | 150 | 31 | 1 | 0 | 0 |
| Epi6 | 1 | IV | 2006-2 / Austr | 21 | 5 | 20 | 5656 | 2185 | 673 | 1510 | 1 | 0 | 0 |
| InterEpi |  |  |  | 11 | 3 | 6 | 1227 | 152 | 58 | 92 | 0 | 0 | 3 |
| Epi7 | 4 | 2a | 2009-2 / Lee | 8 | 5 | 28 | 6803 | 2344 | 1564 | 84 | 0 | 0 | 703 |
| InterEpi |  |  |  | 40 | 5 | 14 | 1707 | 124 | 44 | 0 | 0 | 1 | 80 |
| Epi8 | 3+1 | III+I | 2013-2 / Wind | 18+ | 5 | 23 | 3858 | 1182 | 547 | 510 | 0 | 122 | 0 |
