# Supplementary material for: Long-term persistence of monotypic dengue transmission in small size isolated populations, French Polynesia, 1978-2014
Source: PLoS Negl Trop Dis. 2020 Mar 6;14(3):e0008110. doi: 10.1371/journal.pntd.0008110 (PMC7080275; doi:10.1371/journal.pntd.0008110)
Supplement: S3 Table — (DOCX) [file pntd.0008110.s011.docx]

| **Table S3** Number of confirmed dengue cases by subdivision and epidemic and inter-epidemic period between August 1978 and October 2014 | | | | | | |
| --- | --- | --- | --- | --- | --- | --- |
|  |  |  |  |  |  |  |
|  |  |  |  |  |  |  |
| Period | Windward | Leeward | Australs | Marquesas | Tuamotu-Gambier | % cases in Windward |
| 1979 DENV-4 epidemic | 413 | 17 | 12 | 0 | 2 | 93 |
| 1979-1988 Inter-epidemic | 317 | 4 | 0 | 7 | 1 | 96 |
| 1988/1989 DENV-1 epidemic | 1407 | 139 | 23 | 63 | 3 | 86 |
| 1989/1990 DENV-3 epidemic | 1219 | 45 | 19 | 40 | 3 | 92 |
| 1990- 1996 Inter-epidemic | 914 | 77 | 1 | 21 | 4 | 90 |
| 1996/1997 DENV-2 epidemic | 1522 | 141 | 14 | 56 | 2 | 88 |
| 1997-2000 Inter-epidemic | 133 | 40 | 4 | 7 | 2 | 72 |
| 2001 DENV-1 epidemic | 1036 | 401 | 41 | 34 | 29 | 67 |
| 2001-2006 Inter-epidemic | 148 | 24 | 2 | 7 | 1 | 81 |
| 2006/2007 DENV-1 epidemic | 1467 | 518 | 24 | 144 | 32 | 67 |
| 2007-2009 Inter-epidemic | 129 | 21 | 0 | 0 | 2 | 85 |
| 2009 DENV-4 epidemic | 1714 | 506 | 25 | 49 | 50 | 73 |
| 2009-2010 Inter-epidemic | 86 | 23 | 2 | 7 | 6 | 69 |
| 2013 DENV-1/DENV-3 epidemics | 533 | 522 | 37 | 11 | 79 | 45 |
| TOTAL | 11038 | 2478 | 204 | 446 | 216 |  |
