# Supplementary material for: Long-term persistence of monotypic dengue transmission in small size isolated populations, French Polynesia, 1978-2014
Source: PLoS Negl Trop Dis. 2020 Mar 6;14(3):e0008110. doi: 10.1371/journal.pntd.0008110 (PMC7080275; doi:10.1371/journal.pntd.0008110)
Supplement: S4 Table — (DOCX) [file pntd.0008110.s012.docx]

**Table S4** Air travel between islands in 2010

| ISL1 | ISL2 | PASS_DEP | PASS_ARR | PASS_TOT |
| --- | --- | --- | --- | --- |
| TAHITI | MOOREA | 35458 | 30925 | 66383 |
| TAHITI | BORA-BORA | 6993 | 7255 | 14248 |
| TAHITI | HUAHINE | 3408 | 2389 | 5797 |
| TAHITI | MAUPITI | 578 | 257 | 835 |
| TAHITI | RAIATEA | 6730 | 5710 | 12440 |
| TAHITI | AHE | 329 | 295 | 624 |
| TAHITI | ANAA | 278 | 202 | 480 |
| TAHITI | APATAKI | 170 | 159 | 329 |
| TAHITI | ARATIKA-NORD | 85 | 49 | 134 |
| TAHITI | ARUTUA | 400 | 334 | 734 |
| TAHITI | FAAITE | 124 | 97 | 221 |
| TAHITI | FAKAHINA | 2 | 19 | 21 |
| TAHITI | FAKARAVA | 768 | 596 | 1364 |
| TAHITI | FANGATAU | 47 | 86 | 133 |
| TAHITI | HAO | 435 | 439 | 874 |
| TAHITI | HIKUERU | 62 | 48 | 110 |
| TAHITI | KATIU | 74 | 53 | 127 |
| TAHITI | KAUEHI | 122 | 65 | 187 |
| TAHITI | KAUKURA | 174 | 120 | 294 |
| TAHITI | MAKEMO | 396 | 337 | 733 |
| TAHITI | MANIHI | 421 | 411 | 832 |
| TAHITI | MATAIVA | 288 | 209 | 497 |
| TAHITI | NAPUKA | 66 | 59 | 125 |
| TAHITI | NIAU | 120 | 61 | 181 |
| TAHITI | NUKUTAVAKE | 31 | 45 | 76 |
| TAHITI | PUKA-PUKA | 6 | 6 | 12 |
| TAHITI | PUKARUA | 34 | 21 | 55 |
| TAHITI | RANGIROA | 1980 | 1577 | 3557 |
| TAHITI | RAROIA | 96 | 141 | 237 |
| TAHITI | REAO | 51 | 33 | 84 |
| TAHITI | TAKAPOTO | 165 | 83 | 248 |
| TAHITI | TAKAROA | 300 | 303 | 603 |
| TAHITI | TAKUME | 29 | 0 | 29 |
| TAHITI | TATAKOTO | 54 | 47 | 101 |
| TAHITI | TIKEHAU | 745 | 608 | 1353 |
| TAHITI | MANGAREVA | 332 | 213 | 545 |
| TAHITI | TUREIA | 41 | 34 | 75 |
| TAHITI | VAHITAHI | 25 | 0 | 25 |
| TAHITI | HIVA-OA | 832 | 567 | 1399 |
| TAHITI | NUKU-HIVA | 1460 | 1145 | 2605 |
| TAHITI | RAIVAVAE | 519 | 231 | 750 |
| TAHITI | RIMATARA | 326 | 157 | 483 |
| TAHITI | RURUTU | 978 | 411 | 1389 |
| TAHITI | TUBUAI | 930 | 724 | 1654 |
| BORA-BORA | MOOREA | 329 | 1237 | 1566 |
| BORA-BORA | HUAHINE | 344 | 437 | 781 |
| BORA-BORA | MAUPITI | 83 | 87 | 170 |
| BORA-BORA | RAIATEA | 489 | 652 | 1141 |
| BORA-BORA | FAKARAVA | 3 | 7 | 10 |
| BORA-BORA | RANGIROA | 176 | 0 | 176 |
| BORA-BORA | HIKUERU | 3 | 0 | 3 |
| BORA-BORA | TIKEHAU | 97 | 0 | 97 |
| RAIATEA | MOOREA | 16 | 177 | 193 |
| RAIATEA | HUAHINE | 637 | 633 | 1270 |
| RAIATEA | MAUPITI | 323 | 218 | 541 |
| RAIATEA | RANGIROA | 4 | 0 | 4 |
| RANGIROA | AHE | 23 | 0 | 23 |
| RANGIROA | APATAKI | 39 | 63 | 102 |
| RANGIROA | ARUTUA | 10 | 0 | 10 |
| RANGIROA | FAKARAVA | 191 | 22 | 213 |
| RANGIROA | KAUKURA | 34 | 0 | 34 |
| RANGIROA | HAO | 4 | 0 | 4 |
| RANGIROA | MANIHI | 94 | 10 | 104 |
| RANGIROA | MATAIVA | 35 | 21 | 56 |
| RANGIROA | TAKAPOTO | 16 | 0 | 16 |
| RANGIROA | TAKAROA | 30 | 0 | 30 |
| RANGIROA | TIKEHAU | 122 | 133 | 255 |
| RANGIROA | NUKU-HIVA | 22 | 3 | 25 |
| RANGIROA | NAPUKA | 27 | 0 | 27 |
| RANGIROA | HIVA-OA | 6 | 8 | 14 |
| MOOREA | HUAHINE | 230 | 153 | 383 |
| MOOREA | MAUPITI | 0 | 0 | 0 |
| MOOREA | FAKARAVA | 5 | 3 | 8 |
| MOOREA | TIKEHAU | 0 | 6 | 6 |
| AHE | ARUTUA | 0 | 0 | 0 |
| AHE | MANIHI | 11 | 9 | 20 |
| AHE | TAKAROA | 27 | 26 | 53 |
| ANAA | HIKUERU | 0 | 0 | 0 |
| ANAA | MAKEMO | 28 | 57 | 85 |
| ANAA | RAROIA | 0 | 0 | 0 |
| ANAA | FAKARAVA | 0 | 0 | 0 |
| ANAA | MATAIVA | 3 | 0 | 3 |
| ANAA | TAKAROA | 3 | 0 | 3 |
| APATAKI | ARUTUA | 29 | 12 | 41 |
| APATAKI | FAAITE | 2 | 0 | 2 |
| APATAKI | FAKARAVA | 5 | 3 | 8 |
| APATAKI | MAKEMO | 5 | 0 | 5 |
| APATAKI | NIAU | 0 | 2 | 2 |
| APATAKI | TAKAPOTO | 0 | 5 | 5 |
| ARATIKA-NORD | FAAITE | 3 | 0 | 3 |
| ARATIKA-NORD | KATIU | 0 | 0 | 0 |
| ARATIKA-NORD | KAUEHI | 0 | 1 | 1 |
| ARATIKA-NORD | MAKEMO | 0 | 9 | 9 |
| ARATIKA-NORD | FAKARAVA | 0 | 6 | 6 |
| ARUTUA | KAUKURA | 1 | 0 | 1 |
| ARUTUA | MANIHI | 0 | 0 | 0 |
| ARUTUA | NIAU | 0 | 0 | 0 |
| ARUTUA | FAKARAVA | 0 | 0 | 0 |
| ARUTUA | MATAIVA | 5 | 0 | 5 |
| FAAITE | KATIU | 0 | 0 | 0 |
| FAAITE | MAKEMO | 0 | 38 | 38 |
| FAAITE | FAKARAVA | 0 | 13 | 13 |
| FAAITE | RAROIA | 2 | 0 | 2 |
| FAAITE | TAKAPOTO | 5 | 0 | 5 |
| FAKAHINA | FANGATAU | 39 | 42 | 81 |
| FAKAHINA | HAO | 39 | 36 | 75 |
| FAKAHINA | MAKEMO | 16 | 59 | 75 |
| FAKAHINA | RAROIA | 24 | 0 | 24 |
| FAKAHINA | FAKARAVA | 0 | 6 | 6 |
| FAKAHINA | PUKA PUKA | 0 | 6 | 6 |
| FAKAHINA | TATAKOTO | 6 | 0 | 6 |
| FAKARAVA | HAO | 0 | 1 | 1 |
| FAKARAVA | KATIU | 0 | 0 | 0 |
| FAKARAVA | KAUEHI | 0 | 0 | 0 |
| FAKARAVA | MANIHI | 6 | 27 | 33 |
| FAKARAVA | FANGATAU | 1 | 0 | 1 |
| FAKARAVA | HIKUERU | 0 | 7 | 7 |
| FAKARAVA | NAPUKA | 0 | 6 | 6 |
| FAKARAVA | NIAU | 2 | 0 | 2 |
| FAKARAVA | TIKEHAU | 6 | 0 | 6 |
| FANGATAU | HAO | 21 | 34 | 55 |
| FANGATAU | MAKEMO | 5 | 8 | 13 |
| FANGATAU | NAPUKA | 9 | 0 | 9 |
| FANGATAU | NIAU | 0 | 0 | 0 |
| FANGATAU | RAROIA | 2 | 1 | 3 |
| FANGATAU | TAKUME | 0 | 6 | 6 |
| FANGATAU | TATAKOTO | 0 | 3 | 3 |
| HAO | HAO | 0 | 0 | 0 |
| HAO | KATIU | 0 | 0 | 0 |
| HAO | MAKEMO | 5 | 7 | 12 |
| HAO | NAPUKA | 23 | 0 | 23 |
| HAO | NIAU | 14 | 0 | 14 |
| HAO | NUKUTAVAKE | 28 | 2 | 30 |
| HAO | PUKA-PUKA | 61 | 31 | 92 |
| HAO | PUKARUA | 27 | 0 | 27 |
| HAO | REAO | 51 | 5 | 56 |
| HAO | TAKUME | 1 | 0 | 1 |
| HAO | TATAKOTO | 20 | 15 | 35 |
| HAO | MANGAREVA | 52 | 20 | 72 |
| HAO | TUREIA | 24 | 11 | 35 |
| HAO | VAHITAHI | 2 | 4 | 6 |
| HAO | HIKUERU | 2 | 0 | 2 |
| HIKUERU | MAKEMO | 3 | 9 | 12 |
| HIKUERU | RAROIA | 0 | 0 | 0 |
| HIKUERU | KAUKURA | 3 | 0 | 3 |
| HIVA-OA | NUKU-HIVA | 323 | 382 | 705 |
| HIVA-OA | UA-HUKA | 142 | 165 | 307 |
| HIVA-OA | UA-POU | 261 | 205 | 466 |
| HIVA-OA | MAKEMO | 3 | 0 | 3 |
| KATIU | KAUEHI | 0 | 0 | 0 |
| KATIU | MAKEMO | 17 | 15 | 32 |
| KATIU | NAPUKA | 0 | 0 | 0 |
| KATIU | NIAU | 3 | 0 | 3 |
| KATIU | TAKUME | 0 | 16 | 16 |
| KAUEHI | MAKEMO | 0 | 18 | 18 |
| KAUEHI | NIAU | 0 | 0 | 0 |
| KAUKURA | NIAU | 0 | 0 | 0 |
| MAKEMO | NAPUKA | 5 | 0 | 5 |
| MAKEMO | PUKA-PUKA | 5 | 60 | 65 |
| MAKEMO | RAROIA | 44 | 0 | 44 |
| MAKEMO | TAKUME | 22 | 39 | 61 |
| MANIHI | TAKAPOTO | 10 | 0 | 10 |
| MANIHI | TAKAROA | 17 | 0 | 17 |
| MANIHI | TIKEHAU | 10 | 12 | 22 |
| MAUPITI | MAUPITI | 0 | 0 | 0 |
| NAPUKA | RAROIA | 11 | 0 | 11 |
| NAPUKA | PUKA PUKA | 40 | 2 | 42 |
| NUKU-HIVA | UA-HUKA | 381 | 437 | 818 |
| NUKU-HIVA | UA-POU | 730 | 763 | 1493 |
| NUKU-HIVA | TAKAROA | 0 | 5 | 5 |
| NUKUTAVAKE | TATAKOTO | 3 | 0 | 3 |
| NUKUTAVAKE | TUREIA | 7 | 3 | 10 |
| NUKUTAVAKE | VAHITAHI | 0 | 29 | 29 |
| PUKA-PUKA | RAROIA | 0 | 48 | 48 |
| PUKA-PUKA | TAKUME | 0 | 0 | 0 |
| PUKA-PUKA | REAO | 5 | 0 | 5 |
| PUKARUA | REAO | 8 | 0 | 8 |
| PUKARUA | TATAKOTO | 0 | 7 | 7 |
| PUKARUA | MANGAREVA | 0 | 1 | 1 |
| PUKARUA | VAHITAHI | 6 | 0 | 6 |
| RAIVAVAE | RURUTU | 9 | 3 | 12 |
| RAIVAVAE | TUBUAI | 58 | 83 | 141 |
| RAROIA | TAKUME | 22 | 21 | 43 |
| REAO | TATAKOTO | 0 | 3 | 3 |
| REAO | MANGAREVA | 0 | 9 | 9 |
| REAO | VAHITAHI | 7 | 0 | 7 |
| RIMATARA | RURUTU | 58 | 43 | 101 |
| RIMATARA | TUBUAI | 2 | 0 | 2 |
| RURUTU | TUBUAI | 22 | 48 | 70 |
| TAKAPOTO | TAKAROA | 20 | 11 | 31 |
| TATAKOTO | MANGAREVA | 0 | 5 | 5 |
| MANGAREVA | TUREIA | 0 | 0 | 0 |
| UA-HUKA | UA-POU | 84 | 94 | 178 |

|  |  |  |  |  |  |
| --- | --- | --- | --- | --- | --- |
|  |  |  |  |  |  |
